# Supplementary material for: Patterns of Intron Gain and Loss in Fungi
Source: PLoS Biol. 2004 Nov 30;2(12):e422. doi: 10.1371/journal.pbio.0020422 (PMC532390; doi:10.1371/journal.pbio.0020422)
Supplement: Table S1 — Also available at http://genes.mit.edu/NielsenEtAl/. (4.3 MB ZIP). [file pbio.0020422.st001.zip › NielsenEtAl/html/252.html]

AN6166.1.NCU01322.1.MG06909.1.FG01903.1


```
 CLUSTAL W (1.82) Multiple Sequence Alignments - Introns Inserted


Sequence 1: MG06909.1	418 aa
Sequence 2: FG01903.1	376 aa
Sequence 3: NCU01322.1	353 aa
Sequence 4: AN6166.1	436 aa
Alignment Length: 447 aa
Number Identitical Residues: 89 aa
Alignment Score (without introns) 5526


MG06909.1 	MDLARTLVRSVVRAFYDTKHILVVDALVIHSA~LRDDDLAYLMNMNTKDLHKLCGRLKED
NCU01322.1	MDIAKTLIRCVMRAFYSTQEILIIDALVQHSC~LRDDDLGHLMKLGNKDLHKACAGLRDA
FG01903.1 	MDLAITLVKSVMRAFYQTREILVIDALILHEA2LRDDDLAYLMSINTKDLHKICGKLRED
AN6166.1  	MDLANTLIRTVARSFYETRHILIVDALFIHSV2LHAEDLAFLLGMQQKDLRKLCAKLRED
          	**:* **:: * *:**.*:.**::***. *.  *: :**..*: :  ***:* *. *:: 

MG06909.1 	RFLTV~HTRPELKEGQQRPVNRMYYFIDYRQTIDAIKWRVYTVDKQMQGVTVPADERKEY
NCU01322.1	RFLVV~HTRPELQAGKTRPQNKTYYYIDYRQTIDAIKWRVYKTDKDMQGIAKPSEENKEY
FG01903.1 	RFLVV2HTRSELREGNPRPSNRTWYYIDYRSTIDAIKWRVYTIDKEVQGTTQVASEKKEY
AN6166.1  	RLIAV2NTRAEIRDGSTRPVNREYYYIPLHPVIDAIKYKVSKLTSTIKLQYTPSQERKEY
          	*::.* :**.*:: *. ** *: :*:*  : .*****::* .  . ::     :.*.***

MG06909.1 	FCLRVGCKKEYSLMEVLDKPSARGFLCHDCGSVLKHDPDGGG------GGHKQSTRMNDQ
NCU01322.1	VCPRVGCEAQWSQMEVLDSVSSRGFTCQRCGAVLEQAKEREA------PGHQQLSRMNNQ
FG01903.1 	FCS--HCKAEWTAMEVLDNAGPQGFLCHRCSHVLTFEADRTS------TGHEQSTRLNDQ
AN6166.1  	ICLR--CGAEWTELDVLSLYSEEGFECQNCGAILERTEDVKGSEGIDRTGHEKNSKLMAQ
          	.*    *  ::: ::**.  . .** *: *. :*    :  .:.. .  **:: :::  *

MG06909.1 	FKFITGMLPQIDSVVVPECTFDLAYNAARPVVRDALHQVVASVAVEP-THRQAAVKGLAD
NCU01322.1	FKFMTDMLQEVDKVVIPECNFDLALANARPIVRLETHEVAPSVPVDTALHKPSAVKGLAN
FG01903.1 	FKFISELLPKIDAVHINECDFDRALAKARPVKRDETHQRAQTIAADAGANRPMAVKGLAN
AN6166.1  	LDGMLKLLKQIDSVEIPPNDFDTAWDHKIDVVRN-QHTNPTRAAVVVPSKKQEAVRGNLK
          	:. :  :* ::* * :    ** *      : *   *      ..    ::  **:*  .

MG06909.1 	TGPKSIDINLLSANGPSEADKEAERQRKAKHAEQNALPSWIAQSTISGEAYELDPARTGA
NCU01322.1	TGPKTMQVIISTGDD-DAEQAEAEKKRQEELLRQNALPEWITSSSVP---------TTGG
FG01903.1 	TGPQSIAVNISTSDGLTEAEKAAEQARKEQIAKQNALPSWMSNSTVTGESFSASAPAAGT
AN6166.1  	TDASALEISLTSSEEKSAAEQKEEAARKAALEKQNALPVWHTHSTVSTTAGNVSSIKTEP
          	*...:: : : :.:     :   *  *:    .***** * : *::.  : . ..  :  

MG06909.1 	VAGAIASTTTTETKKDAMDAKDHAEIDDYFAKLAKEQEAEAARKAAEAEEEDDDDDEDDD
NCU01322.1	FK---------GSGPAAASLLDSSSAVKDEAVVKDEP---PAKKVKMEIQGDISVKDEE-
FG01903.1 	TP--IIKKEGKDSESAAAPAAN-AQIDDIFEMIKAEQ---AAKQAQVESEEDDDDDEDE-
AN6166.1  	DVKIKPELLKEEEDQKPSLVDLDDKVAAYYAEMEREKALQAQEDASSAEDSDEFDEFEDV
          	      .         .       .       :  *   .. ...    : *   . :: 

MG06909.1 	GMFEDVVAGG---------------------TPAVETPKSSLKREASSGSEGKPAKKVKV
NCU01322.1	----------------------------------------------------HMPMSFKM
FG01903.1 	--FEDV-----------------------------------------------PAKRVKI
AN6166.1  	GVSASASPAVGGVNGADPTRAPSGIKRELDTDSGTSAPQTATGTPSTPADEGPAAKKIKT
          	.   .  .. .. ..:..: :.:. . . .: .. .:..::    ::.....  .  .* 

MG06909.1 	EEPSPTVNAAADAAGGDESDDEIEFEDV-
NCU01322.1	EG-------------GDEEEDDLEFEDVM
FG01903.1 	EH-----EVKKEEDDGEDSD-EIEFEDV-
AN6166.1  	ELEN-------EVKKEESDEDDEEFEDV-
          	*  .       .    :..:.: *****
```
